# Supplementary material for: Controlling single rare earth ion emission in an electro-optical nanocavity
Source: Nat Commun. 2023 Mar 28;14:1718. doi: 10.1038/s41467-023-37513-w (PMC10049985; doi:10.1038/s41467-023-37513-w)
Supplement: Supplementary file 1 — Supplementary Information [file 41467_2023_37513_MOESM1_ESM.pdf]

Supplementary Information for "Controlling single rare earth ion emission in an electro-optical nanocavity"

Likai Yang,[1] Sihao Wang,[1] Mohan Shen,[1] Jiacheng Xie,[1] and Hong X. Tang[1,*]

[1]*Department of Electrical Engineering,*

Yale University, New Haven, CT 06511, USA

[*] hong.tang@yale.edu

SUPPLEMENTARY NOTE 1. DEVICE FABRICATION

The fabrication process of our devices is described in Supplementary Fig. 1. We start with a 600 nm erbium (Er) doped lithium niobate on insulator (LNOI) film, with 2 µm silicon dioxide (SiO_2) as substrate. It is first thinned down to 300 nm by a reactive ion etching (RIE) process with argon (Ar) plasma. The waveguide is then defined by electron beam lithography (EBL), using hydrogen silsesquioxane (HSQ) as resist. Another Ar plasma RIE etches 180 nm into LN to form a ridge waveguide. The waveguide width is set to be 1.2 µm and the angle of etching process is $\sim$60°. After etching, the residue resist is removed by buffered oxide etch (BOE). The second EBL defines photonic crystal holes and the slab with HSQ. The hole dimensions are 600 nm$\times$350 nm. The EBL dose is carefully adjusted so that the actual dimensions of the holes match well with designed values. After that, a RIE process etches through LN. Removal of residue resist with BOE will cause small undercut in the SiO_2 layer, but the device structure is robust enough for further fabrication. The third EBL uses polymethyl methacrylate (PMMA) to lift off metal electrodes, which consist of 5 nm chromium (Cr) under 50 nm gold (Au) deposited by thermal evaporation. The thin Cr layer is used to improve the adhesion. The gap between metal electrodes is designed to be 5 µm so that the optical mode will not be perturbed. We do not see noticeable difference in optical quality factor with or without the metal electrodes. Finally, the chip is dipped into BOE for a longer time to form a suspended structure.

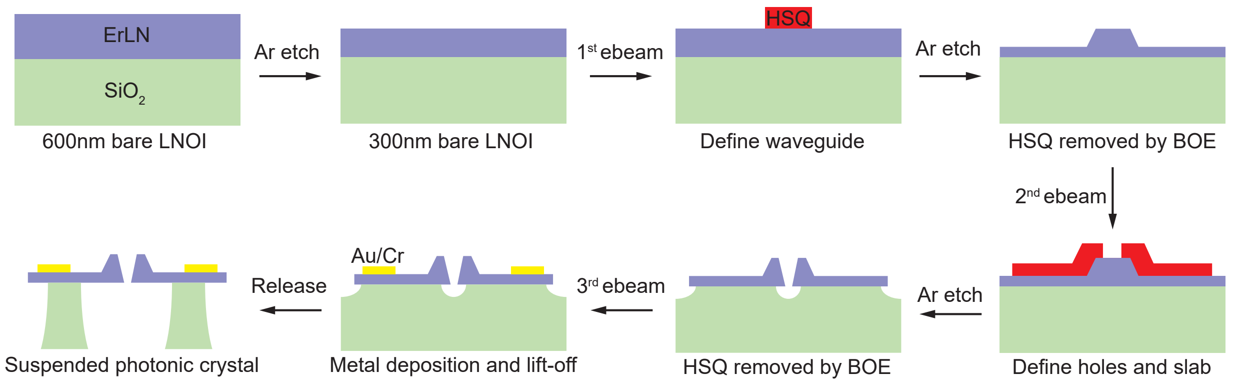

Supplementary Fig. 1. **Fabrication process for photonic crystal devices.**

SUPPLEMENTARY NOTE 2. MEASUREMENT SETUP

The schematic drawing of our measurement setup is shown in Supplementary Fig. 2. Light from a tunable laser (Santec TSL-710) is chopped by acousto-optic modulators (AOM) to generate

excitation pulses. In the case of frequency sweeping, the internal piezoelectric tuning function of the laser is utilized. On-off extinction ratio of >100 dB is reached using 2 AOMs. The light is then sent to our device under test (DUT) mounted at 1 K plate of a dilution refrigerator. The reflection as well as fluorescence signal from DUT is collected via an optical circulator and sent to a superconducting nanowire single photon detector (SNSPD), sitting at 200 mK of the same fridge. Pulsed electro-optic tuning voltage is generated by a high voltage amplifier. The amplifier has a bipolar maximal output voltage of 200V and bandwidth ∼1 MHz. The bias circuit of the SNSPD consists of a pulse generator and an in-line 50 kΩ resistor. This allows the SNSPD to be turned off during excitation pulse to prevent saturation, and turned on in the fluorescence collection window. Readout of SNSPD signal is done by a pulse counter connected to PC. The pulse counter (Picoharp 300) works in a real-time data collection mode, in which the time tag of each detected photons can be registered. The AOM, tuning voltage, and SNSPD bias pulses are all synchronized with controlled delay and width.

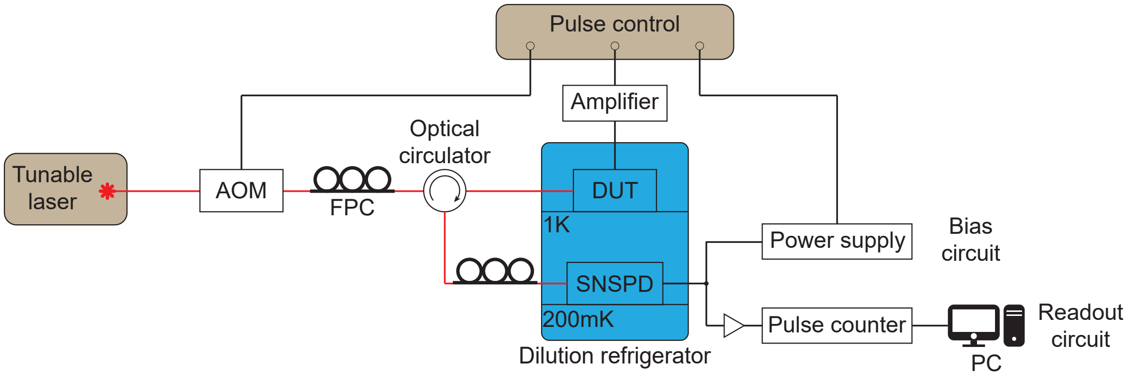

Supplementary Fig. 2. **Schematic drawing of measurement setup.** AOM: acousto-optic modulator. FPC: fiber polarization controller. DUT: device under test. SNSPD: superconducting nanowire single photon detector.

SUPPLEMENTARY NOTE 3. PURCELL ENHANCEMENT

Theoretically, the Purcell enhancement of an Er ion inside a cavity can be expressed as [1]

$$P(\vec{r}) = \frac{3}{4\pi^2}\frac{\beta Q\lambda^3}{\chi_L n^3 V_{\text{mode}}}\frac{|E(\vec{r})|^2}{|\max(E(\vec{r}))|^2}, \tag{1}$$

where $\beta = 0.22$ [2] is the branching ratio of radiative transition of ErLN, $n \approx 2$ is the cavity refractive index, and $\chi_L = [(n^2+2)/3]^2 \approx 2$ is the local field correction. The mode volume is

defined as $V_{\text{mode}} = \frac{\int \varepsilon |E(\vec{r})|^2 d\vec{r}}{\max(\varepsilon |E(\vec{r})|^2)}$, with integral over all space. The Purcell enhancement of an ion depends on the field strength $|E(\vec{r})|$ at the ion location. The average Purcell enhancement of the cavity is then the average over all ions, weighted by their field intensity:

$$P_{\text{avg}} = \frac{\int_{\text{LN}} P(\vec{r}) |E(\vec{r})|^2 d\vec{r}}{\int_{\text{LN}} |E(\vec{r})|^2 d\vec{r}} = \frac{3}{4\pi^2} \frac{\beta Q \lambda^3}{\chi_L n^3 V_{eff}}. \tag{2}$$

Here, the effective mode volume V_{eff} can be expressed as

$$V_{\text{eff}} = \frac{\int |E(\vec{r})|^2 d\vec{r} \int_{LN} |E(\vec{r})|^2 d\vec{r}}{\int_{LN} |E(\vec{r})|^4 d\vec{r}}. \tag{3}$$

This can be calculated from finite element simulation of the cavity mode profile, yielding $V_{\text{eff}} = 2\,\mu\text{m}^3$. This gives $P_{\text{avg}} = 150$.

The distribution of Purcell factor for ions in the cavity can also be extracted using these equations. We calculate the percentage of ions in the cavity that has Purcell factor larger than different P_{min}. The results are shown in Supplementary Fig. 3.

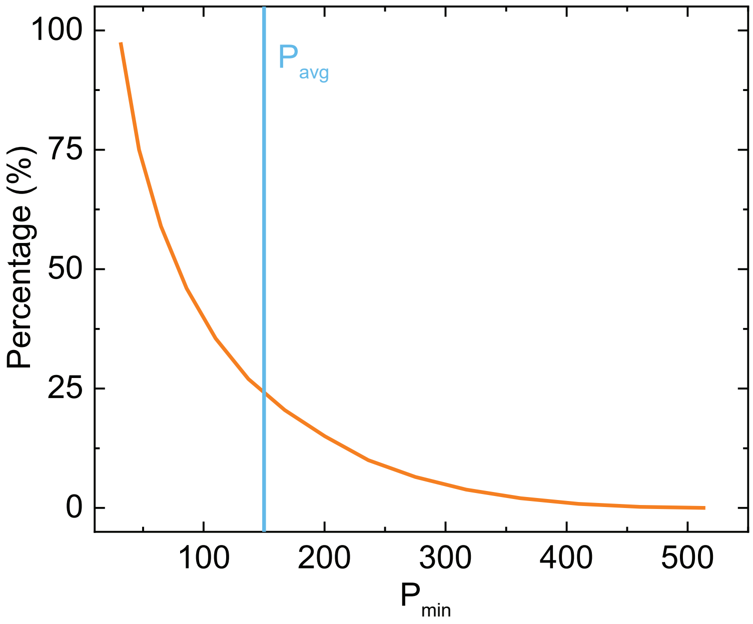

Supplementary Fig. 3. **Distribution of Purcell enhancement for ions in the cavity.** The y axis denotes the percentage of ions that has Purcell factor larger than P_{min}, with respect to all ions in a volume $V_{\text{eff}} = 2\,\mu\text{m}^3$.

SUPPLEMENTARY NOTE 4. SINGLE ION COUNT RATE AND $g^{(2)}$ ANALYSIS

Theoretically, the count rate we get from a single Er ion in the cavity can be calculated as

$$N = P_e \times P_{\text{decay}} \times P_{\text{cav-wg}} \times P_{\text{fiber-chip}} \times P_{\text{loss}} \times \eta_{\text{SNSPD}} \times \frac{1}{T_{\text{rep}}}. \qquad (4)$$

Here, $P_e = 1/2$ is the maximum probability an ion being in excited state after an incoherent pump. $P_{\text{decay}} = 1 - e^{t/T_1}$ is the probability it decays to ground state during the collection window t. For us, $t = 10\,\mu$s and $T_1 = 10\,\mu$s, so $P_{\text{decay}} = 0.63$. $P_{\text{cav-wg}} = \frac{\kappa_{\text{ex}}}{\kappa_{\text{ex}}+\kappa_{\text{in}}} \approx 1/2$ is the coupling rate between cavity and waveguide. $P_{\text{fiber-chip}} = 0.1$ is the single side fiber-to-chip coupling efficiency. Loss in optical components such as the circulator and the fibers are included in $P_{\text{loss}} \approx 0.6$. Collection efficiency of our SNSPD is $\eta_{\text{SNSPD}} = 50\,\%$. These account for the number of photons detected after each single excitation pulses. It is then multiplied by the repetition rate $1/T_{\text{rep}} = 50\,\text{kHz}$ to get the actual count rate. The resulting value is 236 Hz, in rough agreement with the $\sim$160 Hz we get from our measurement.

The photons we collect for second-order correlation measurement are from two parts, the single ion I_{ion} and the background I_{bg}. The background is attributed to the emission from ions weakly coupled to the cavity and the dark count from SNSPD. Here, we take $g^{(2)}_{\text{ion}}(0) = \langle I^2_{\text{ion}}\rangle/\langle I_{\text{ion}}\rangle^2 = 0$ and $g^{(2)}_{\text{bg}}(0) = \langle I^2_{\text{bg}}\rangle/\langle I_{\text{bg}}\rangle^2 = 1$. The signal-to-noise ratio is defined as $\text{SNR} = \langle \text{I}_{\text{ion}}\rangle/\langle \text{I}_{\text{bg}}\rangle$. Then, the measured second-order autocorrelation function would be

$$g^{(2)}(0) = \frac{\langle I^2\rangle}{\langle I\rangle^2} = \frac{\langle (I_{\text{ion}}+I_{\text{bg}})^2\rangle}{\langle I_{\text{ion}}+I_{\text{bg}}\rangle^2} = \frac{\langle I^2_{\text{ion}}\rangle + \langle I^2_{\text{bg}}\rangle + 2\langle I_{\text{ion}}\rangle\langle I_{\text{bg}}\rangle}{\langle I_{\text{ion}}\rangle^2 + \langle I_{\text{bg}}\rangle^2 + 2\langle I_{\text{ion}}\rangle\langle I_{\text{bg}}\rangle} = \frac{2\text{SNR}+1}{(\text{SNR}+1)^2}. \qquad (5)$$

For us, we get $g^{(2)}(0) = 0.38$. This gives SNR = 3.70, suggesting that $\sim$79 % of the collected photons are from a single ion. This is in agreement with our estimation of $\sim$160 Hz single ion count rate and $\sim$40 Hz background count rate. Along with the fact that the emission peaks do not split with spectral diffusion, it can be confirmed that majority of photons come from a single emitter.

Apart from device optimization to increase Purcell enhancement, improvement of $g^{(2)}(0)$ can mainly come from two aspects. The first is improving fiber-to-chip coupling efficiency. With optimized fiber glue process and better grating coupler design, a single-side transmission of 50 % is achievable. This will increase single ion count rate to $\sim$1000 Hz. The other factor lies in suppression of background Er emission. This can be done by using smaller doping concentration or tuning the cavity frequency further away. The ultimate background is the dark count from

SNSPD, which is $\sim$20 Hz in our case. Implementing these improvements will result in $g^{(2)}(0) \approx 0.04$.

SUPPLEMENTARY NOTE 5. THEORETICAL ESTIMATION OF DC STARK SHIFT

The DC stark effect of $4f$-$4f$ transitions of Er ions in lithium niobate has been measured in previous literature to be 25 kHz/V$\cdot$cm^{-1} [3] for electric field along crystal z-direction and vanish for electric field perpendicular to z-direction [4]. In our device, the tuning electric field is applied mostly along crystal y-direction so that the DC stark shift is minimized. However, the non-zero z-component of electric field might still cause small shift of Er transition frequency. Supplementary

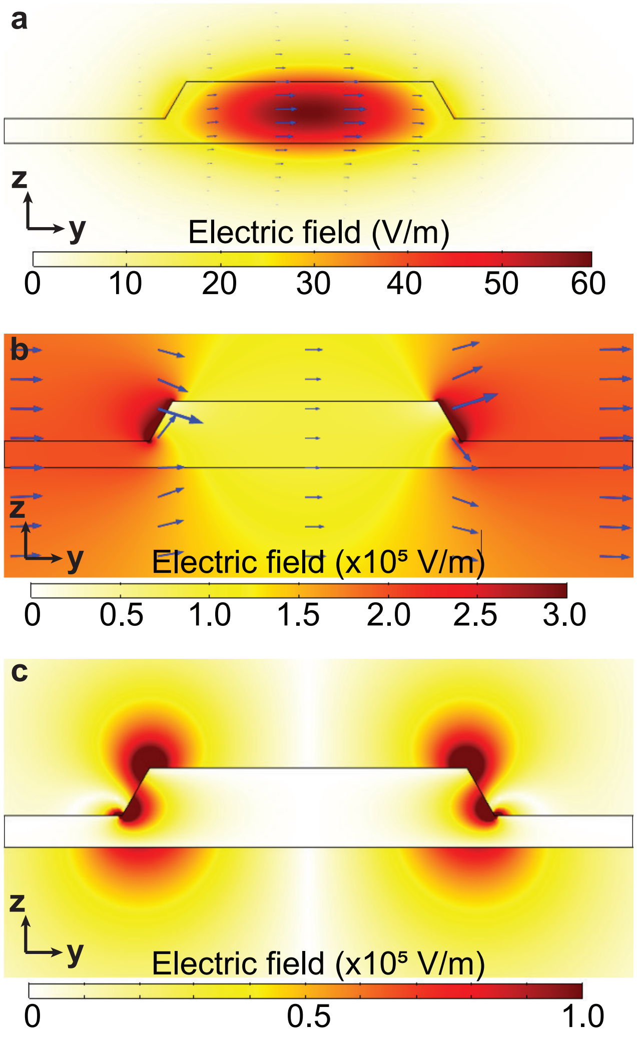

Supplementary Fig. 4. Electric field distribution in the lithium niobate waveguide for a. optical mode; b. 1 V DC tuning voltage; c. z-component field for 1 V DC tuning voltage. The blue arrows indicate the field direction.

Fig. 4 shows the simulated electric field distribution for: a. optical mode; b. 1 V DC tuning voltage; c. z-component (E_z) field for 1 V DC tuning voltage. Gemeotry same with our devices is used in the simulation, where the film thickness is 300 nm, the waveguide width is 1.2 µm, the slab thickness is 120 nm, and the gap between metal electrodes is 5 µm. From Fig. 2c we can see that the electric field component E_z for 1 V tuning voltage ranges from 0 at the center of the waveguide to $\sim 5\times10^4$ V/m at the edge. This will result in a non-zero DC stark shift of $\sim$10 MHz/V for ions that are on the edge, which also have smaller Purcell enhancement. The DC stark shift should decrease toward zero for ions in the center with larger Purcell enhancement. Still, the value 10 MHz/V is an order of magnitude smaller than the electro-optic tuning rate of $\sim$200 MHz/V.

---

[1] Miyazono, E., Craiciu, I., Arbabi, A., Zhong, T. & Faraon, A. Coupling erbium dopants in yttrium orthosilicate to silicon photonic resonators and waveguides. *Optics express* **25**, 2863–2871 (2017).

[2] McAuslan, D., Longdell, J. J. & Sellars, M. Strong-coupling cavity qed using rare-earth-metal-ion dopants in monolithic resonators: What you can do with a weak oscillator. *Physical Review A* **80**, 062307 (2009).

[3] Hastings-Simon, S. R. *et al.* Controlled stark shifts in er3+-doped crystalline and amorphous waveguides for quantum state storage. *Optics communications* **266**, 716–719 (2006).

[4] Wong, K.-K. *Properties of lithium niobate*. 28 (IET, 2002).
